# Who, where, what and where to now? A snapshot of publishing patterns in Australian orthopaedic surgery

**DOI:** 10.1111/ans.14177

**Published:** 2017-08-15

**Authors:** Nicholas Croker, Abhirup Lobo, Anne Croker, Zsolt J. Balogh, David Dewar

**Affiliations:** ^1^ Department of Orthopaedics John Hunter Hospital Newcastle New South Wales Australia; ^2^ Department of Rural Health University of Newcastle Newcastle New South Wales Australia; ^3^ Department of Traumatology John Hunter Hospital Newcastle New South Wales Australia; ^4^ School of Medicine and Public Health University of Newcastle Newcastle New South Wales Australia

**Keywords:** Australia, orthopaedics, research, surgery, training

## Abstract

**Background:**

Development of core research competency is a principle of orthopaedic surgical training in Australia. This paper aims to provide an objective snapshot of publications by Australian orthopaedic trainees and surgeons, to contribute to the discussion on how to identify and build on research capability in the Australian Orthopaedic Association (AOA).

**Methods:**

By analysing journals with a journal impact factor >1 from 2009 to 2015, data were gathered to explore scientific journal publications by Australian orthopaedic surgeons and trainees in relation to who are the authors, what they are reporting and where they are publishing.

**Results:**

One thousand five hundred and thirty‐nine articles were identified with 134 orthopaedic trainees and 519 surgeons as authors. The publication rate for both trainees and surgeons was just over two in five. The majority of studies were of level three or four evidence (Oxford's Centre for Evidence‐Based Medicine guidelines). Only 5% of trainee papers were published without surgeons’ co‐authorship. Eighty‐six percent of papers published by surgeons did not involve a trainee. The rates of trainees publishing with other trainees were low.

**Conclusion:**

Only 5% of trainee papers were published without surgeons' co‐authorship, highlighting the importance of surgeon mentorship in developing trainee research capability. The 86% of papers published by surgeons without trainee co‐authorship raises the question of missed mentoring opportunities. Low rates of trainee co‐authorship highlight potential for trainees to work together to support each other's research efforts. There is scope for more studies involving higher levels of evidence. This paper raises discussion points and areas for further exploration in relation to AOA trainee research capability.

## Introduction

Research is fundamental to ensure high‐quality, accessible patient‐centred orthopaedic surgical services for the Australian community. Accordingly, there is an impetus to develop research capability within orthopaedics.^1^ Developing research capability is a challenging undertaking requiring widespread multifaceted engagement from the Australian orthopaedic surgical community.

The Australian Orthopaedic Association (AOA) promotes the development of research capability through provision of funding, seminars and requirements in training selection and completion.^2^, ^3^, ^4^ To fulfil training requirements, Australian orthopaedic trainees must provide evidence of dissemination of research findings through conference presentations or journal publications. Journal publications have the potential for a wider audience and greater impact on clinical practice.

The development of a researcher's knowledge, behaviours and attitudes is a lifelong pursuit, requiring a supportive environment, mentorship and guidance, as well as preparedness, motivation and ability to work with others.^5^ The development of research capability can be guided by models such as the ‘Bland *et al*. 2002 Model of Faculty Research Productivity’, subjectively evaluated using models such as the ‘Vitae Researcher Development Framework’ or objectively measured via tracking the dissemination of individual's findings through citation‐based metrics.^5^, ^6^ In relation to the latter, journal impact factor (JIF) is a dynamic bibliometric measure of journal prestige calculated by averaging the citation per article published in the journal over the prior two calendar years.^7^ While a rudimentary marker, it is widely recognized as a reasonable proxy indicator of journal quality.^8^, ^9^ Additional surrogate measures include ‘levels of evidence’, which have been adopted from Oxford's Centre for Evidence‐Based Medicine (CEBM) by several orthopaedic journals.^10^, ^11^ While research collaboration is complex and difficult to measure, co‐authorship provides a useful surrogate measure of researchers’ collaboration.^12^

Currently, there are no objective data to document the current state of orthopaedic research in Australia. We aim to provide an objective snapshot of research publications in orthopaedic journals with a JIF >1 by Australian orthopaedic trainees and surgeons, in order to contribute to the ongoing discussion on building research capability in the AOA.

## Methods

The method of scoping and analysing publishing patterns was designed to answer the question: What are Australian orthopaedic surgeons and trainees publishing, in relation to *who* are the authors (trainees and surgeons), *what* they are reporting (levels of evidence for clinical practice) and *where* are the articles published (impact factor of the journal)?

A list of trainees was obtained from the AOA website. A list of Australian orthopaedic surgeons was compiled using the ‘Find a Surgeon’ tool on the AOA website. Two independent reviewers completed a Scopus search to collect all articles published by each individual on the list. Letters, opinion pieces, abstract‐only entries and articles published in a journal with JIF <1 were excluded. Data were extracted from each article, including Journal, Title and Year. Journal articles were reviewed for level of evidence (using Oxford's CEBM guidelines), type of study and impact factors of the publishing journal.^13^ The position of the author (first author, middle author and last author) was recorded. Data were analysed to determine patterns of trainee and surgeon publication including co‐authorship, JIF and level of evidence. Microsoft Excel (2016, Version 1706; Microsoft Corporation, Redmond, WA, USA) was used for statistical analysis by using calculations on spreadsheets and the ‘Data Analysis’ functions.

## Results

One thousand five hundred and thirty‐nine articles published across 274 different journals satisfied the inclusion criteria. Analysis of the 1539 articles identified 134 orthopaedic trainees and 519 orthopaedic surgeons as authors (Table 1). The rates of trainees and surgeons publishing were similar (Table 1). Sixty percent of manuscripts were published in journals with a primary focus on orthopaedics; however, there was a wide selection of non‐orthopaedic journals where manuscripts were published (Table 2).

**Table 1:** Australian orthopaedic surgeons and trainees as authors

	First	Middle	Last	Total authorships	Total individuals	%
Trainees	110	111	16	237	134	44 (134/306†)
Surgeons	236	1058	677	1971	519	45 (519/1143‡)
						*P* = 0.77§

† As census data were not accessible for 2009–2010 trainees, these years were assigned a total based on the average of 2011–2015.

‡ The total number of surgeons was averaged over 6 years based on the Royal Australasian College of Surgeons census data.

§ Statistical significance set as *P* < 0.05 (Fisher's exact test).

**Table 2:** Journal type utlilized for publication

	Number of journals utilized	Paper in journals
Orthopaedic journals	63 (23%)	928 (60%)
Non‐orthopaedic journals	211 (77%)	611 (40%)

Orthopaedic surgeons' articles compared to trainees' articles were published in journals with a higher JIF. A statistically significant difference between median JIF for trainee and surgeon authorships was identified (using a two‐tailed Mann–Whitney *U*‐test) (Table 3).

**Table 3:** JIF of papers with Australian orthopaedic surgeons and trainees as authors

	Median JIF	Interquartile range
Surgeons	2.66	1.64
Trainees	1.94	1.58
		*P* < 0.001†

† Statistical significance set as *P* < 0.05 (Mann–Whitney *U*‐test).

JIF, journal impact factor.

The majority of studies were clinical. Clinical studies were predominantly of level three or four evidence according to Oxford's CEBM guidelines (Table 4).

**Table 4:** Types of articles (including Oxford's Centre for Evidence‐Based Medicine levels of evidence for clinical papers)

Study type	Surgeons' authorships	Trainees' authorships	Papers
Type 1	82 (4%)	22 (9%)	62 (4%)
Type 2	159 (8%)	34 (14%)	123 (8%)
Type 3	437 (22%)	48 (20%)	291 (19%)
Type 4	644 (33%)	80 (34%)	523 (34%)
Type 5	164 (8%)	10 (4%)	130 (8%)
Basic science (B)	261 (13%)	15 (6%)	229 (15%)
Biomechanical (M)	198 (10%)	21 (9%)	157 (10%)
Surveys (S)	26 (1%)	7 (3%)	24 (2%)
Total	1971	237	1539

There were low rates of trainees publishing with other trainees. Comparatively, rates of surgeons’ co‐authoring articles with other surgeons/trainees were higher (Table 5). Trainees published almost exclusively with a surgeon as co‐author (Table 5).

**Table 5:** Publication co‐authorship

	Trainee	Surgeon
Papers with one …	195 (13%)	1087 (71%)
Papers with two or more …	20 (1%)	380 (25%)
Papers with no …	1324 (86%)	72 (5%)
Total	1539	1539

## Discussion

Forty‐five percent of Australian orthopaedic surgeons and 44% of Australian orthopaedic trainees have published in a journal with a JIF of >1 between 2009 and 2015. Given the competitive nature of research publication, achieving this rate is an impressive statistic. Additionally, the 211 non‐orthopaedic journals that were utilized for publication reflects the considerable breadth of research being undertaken by orthopaedic trainees and surgeons. Trainee publishing patterns seem to be reflecting those of the surgeons both in rates of publishing in these journals and types of studies produced. This provides promising indication that surgeon mentorship is having an impact on trainee publication tendencies. While training requirements undoubtedly provided impetus for trainees’ publications, further research is needed to determine personal and organizational factors that facilitated the research undertaken by Australian orthopaedic trainees and surgeons.

The distribution of levels of evidence also suggests scope for an increased number of studies involving higher levels of evidence. Higher levels of evidence tend to require prospective clinical trials. If the AOA is to promote the development of research capability through participation in such studies, a number of areas need to be addressed. These areas include time taken to complete the trial, appropriate research training, supervision, mentorship and creating a research‐nourishing environment with supportive infrastructure.^14^

In relation to developing trainee research capability, there are certain figures that stand out in the analysis that point to opportunities for future efforts. Only 5% of trainee papers were published without surgeons’ co‐authorship, perhaps reflecting the importance of mentorship in developing trainee research capability. Eighty‐six percent of papers published by surgeons did not involve a trainee, raising the question of missed mentoring opportunities. Only 1% of papers involving a trainee involved more than one trainee, which highlights potential for trainees to work together in support of each other's research efforts. One benefit of this cooperation would be expedited data collection. More importantly, collaboration on the conceptualization of multiple studies would be a valuable strategy to increase research quality, productivity and subsequently researcher capability, all within the constraints of their allotted time frame. The ‘trainee collaboratives’ in the UK have reported success in this regard.^15^, ^16^, ^17^ Key intentions behind these collaboratives that are translatable to other contexts, including Australian orthopaedic surgery research, are captured in the following insights: ‘Trainees are ideally placed to deliver this model; they follow a rotational pattern through several hospitals, are in regular contact with each other, are motivated and require formalized evidence of research and audit. As these trainees become consultants, a culture of trials could be distilled in UK surgical practice’.^18^ Such translatability is supported by the recent use of surgical trainee collaboratives in Europe and recognition that collaborative research networks play an important role in supporting clinical research in orthopaedic surgery.^19^, ^20^ However, care must be taken to ensure collaboration involves contribution to conceptualization, as data collection whilst essential does not necessarily promote holistic development of an individual's research capability.

Within this international support for surgical trainee collaboratives and clinical research in orthopaedics, there is scope for further exploration of the possibility of utilizing trainee collaborative research networks within the AOA network of trainees. Such networks could be the context for mentored, prospective, multicentre clinical trials. Important to the development of such trials is recognition of the multifaceted nature of research capability and the importance of providing appropriate leadership support.^14^ In recognizing the multifaceted nature of research capability, attention needs to be given to developing trainees’ research attributes, supporting their surgeon mentors and ensuring appropriate resources. Viewing the development of research capability as involving social capital (researchers’ network ties and linkages) and human capital (researchers’ personal attributes developed through education and training) enables the complexity of research skills to be embraced and the relationships involved in mentoring them valued.^12^

### Limitations

As co‐authorship was used as a surrogate measure of collaboration, the nature of individual contributions to publications could not be identified. Without knowledge of authors’ contribution to the research and the publication, the rates of actual collaboration may have been overestimated.

This article does not attempt to quantify the breadth or focus of Australian orthopaedic research, but rather provides an objective snapshot of what is being published by whom in relation to levels of evidence. The snapshot was undertaken to facilitate discussion about identifying and building on research capability in the AOA. Such discussion should acknowledge the complexity of orthopaedic surgery research and the value of researchers working together to produce quality research outputs and develop research capability within the AOA.

### Areas requiring further exploration

Exploring the nature of relationships in research collaborative networks is an area for future research. Other areas for further exploration arising from our research include:

1. exploration of orthopaedic trainees’ and surgeons’ experiences with developing their research capability;
2. exploration of orthopaedic surgeons’ experiences with mentoring the development of trainees’ research capability;
3. examination of time taken from inception of a study to publication, to identify reasonable expectations for level of research capability by an orthopaedic trainee and demonstration of such capability; and
4. analysis of the extent to which publishing as a trainee results in commitment to research and further publications as a surgeon.

## Conflicts of interest

None declared.
